# Speech motor brain regions are differentially recruited during perception of native and foreign-accented phonemes for first and second language listeners

**DOI:** 10.3389/fnins.2014.00275

**Published:** 2014-09-03

**Authors:** Daniel Callan, Akiko Callan, Jeffery A. Jones

**Affiliations:** ^1^Center for Information and Neural Networks, National Institute of Information and Communications Technology, Osaka UniversityOsaka, Japan; ^2^Multisensory Cognition and Computation Laboratory Universal Communication Research Institute, National Institute of Information and Communications TechnologyKyoto, Japan; ^3^Laurier Centre for Cognitive Neuroscience and Department of Psychology, Wilfrid Laurier UniversityWaterloo, ON, Canada

**Keywords:** speech perception, accent, fMRI, Broca's area, premotor, cerebellum, internal model, non-native speech

## Abstract

Brain imaging studies indicate that speech motor areas are recruited for auditory speech perception, especially when intelligibility is low due to environmental noise or when speech is accented. The purpose of the present study was to determine the relative contribution of brain regions to the processing of speech containing phonetic categories from one's own language, speech with accented samples of one's native phonetic categories, and speech with unfamiliar phonetic categories. To that end, native English and Japanese speakers identified the speech sounds /r/ and /l/ that were produced by native English speakers (unaccented) and Japanese speakers (foreign-accented) while functional magnetic resonance imaging measured their brain activity. For native English speakers, the Japanese accented speech was more difficult to categorize than the unaccented English speech. In contrast, Japanese speakers have difficulty distinguishing between /r/ and /l/, so both the Japanese accented and English unaccented speech were difficult to categorize. Brain regions involved with listening to foreign-accented productions of a first language included primarily the right cerebellum, left ventral inferior premotor cortex PMvi, and Broca's area. Brain regions most involved with listening to a second-language phonetic contrast (foreign-accented and unaccented productions) also included the left PMvi and the right cerebellum. Additionally, increased activity was observed in the right PMvi, the left and right ventral superior premotor cortex PMvs, and the left cerebellum. These results support a role for speech motor regions during the perception of foreign-accented native speech and for perception of difficult second-language phonetic contrasts.

## Introduction

A growing body of research suggests that speech motor areas are recruited to facilitate auditory speech perception when the acoustic signal is degraded or masked by noise (Callan et al., 2010; Schwartz et al., 2012; Adank et al., 2013; Moulin-Frier and Arbib, 2013). Researchers hypothesize that auditory speech signals are translated into internally simulated articulatory control signals (articulatory-auditory internal models), and that these internal simulations help to constrain speech perception (Callan et al., 2004a; Wilson and Iacoboni, 2006; Skipper et al., 2007; Iacoboni, 2008; Poeppel et al., 2008; Rauschecker, 2011; Schwartz et al., 2012). Indeed, brain imaging studies have demonstrated that activity increases in speech motor areas when participants listen to speech in noise relative to when they listen in noise-free conditions (Callan et al., 2003a, 2004b). Increased activity in speech motor areas has also been observed when listeners identify phonetic categories that are not in their first language (non-native), relative to the activity observed when they identify phonetic categories from their first language (native) (Callan et al., 2003b, 2004a, 2006a; Wang et al., 2003). Moreover, activity in speech motor areas has been found to increase when participants listen to sentences in their first language when they are spoken in an unfamiliar accent (Adank et al., 2013). These observations, as well as observations from other studies that have demonstrated that speech motor brain regions are responsive to both production and perception of speech, support motor simulation theories of speech perception (Callan et al., 2000, 2006b, 2010; Wilson et al., 2004; Nishitani et al., 2005; Meister et al., 2007). In this study, we investigated the neural processes involved in the perception of phonetic categories from one's first language produced by native speakers, as well as those produced by speakers with a foreign-language accent. We compared the neural activity in these conditions to the activity observed when participants perceived phonetic categories from their second language (again, both produced by a native speaker of that second language, and produced by a speaker with a foreign-language accent).

Adults often have considerable difficulty discriminating and identifying many non-native phonetic categories in their second language that overlap with a single phonetic category in their first (native) language, even after years of exposure to that second language (Miyawaki et al., 1975; Trehub, 1976; Strange and Jenkins, 1978; Werker et al., 1981; Werker and Tees, 1999). The English /r/ and /l/ phonetic contrast is an example of a difficult non-native phonetic contrast for native Japanese speakers (Miyawaki et al., 1975). Intensive phonetic identification training can result in long-term improvement in speech perception that generalizes to novel stimuli (Lively et al., 1994; Akahane-Yamada, 1996; Bradlow et al., 1999). Perceptual identification training can also lead to improvements in production (Bradlow et al., 1997), even in the absence of formal production training. The observation that perceptual improvements lead to production improvements suggests that a perceptual-motor component may be responsible for the improved phonetic identification. Indeed, several brain-imaging studies support the hypothesis that neural processes associated with speech production constrain and facilitate phoneme identification (Callan et al., 2004a, 2010; Skipper et al., 2007).

Similar to the difficulties listeners have discriminating and identifying non-native phonetic contrasts in a second language, foreign-accented native speech is often difficult for a native speaker of the language to perceive (Goslin et al., 2012; Adank et al., 2013; Moulin-Frier and Arbib, 2013). Recent evidence suggests that speech motor processes are recruited to facilitate perception when listening to foreign-accented productions of a language (Adank et al., 2013; Moulin-Frier and Arbib, 2013). For example, Adank et al. (2013) found evidence for sensorimotor integration during processing of foreign-accented speech when they asked one group of participants to imitate the unfamiliar foreign-accent of a speaker who uttered sentences in the participants' first language, and compared their brain activity to another group of participants who repeated the same sentences in their own native accent. Adank et al. (2013) compared the levels of activation in the speech motor regions of the brain (including the inferior frontal gyrus, and Broca's area) when participants listened to sentences before a production task, to the levels of activation observed when participants listened to sentences after a production task. Larger differences in speech motor activity were observed for the participants who imitated the unfamiliar, foreign-accented speech, compared to the participants who repeated the sentences in their own accent, specifically when the participants listened to the sentences before compared to after the production task.

The goal of the present study was to differentiate the neural processes that are involved in the perception of phonetic categories in a second language (non-native), from the neural processes involved in the perception of foreign-accented productions of phonetic categories from one's first language. In this study, native English (Eng) and Japanese (Jpn) speakers listened to native English (“unaccented”) and Japanese (“accented”) productions of English syllables that began with either /r/ or /l/. The Japanese productions of the English syllables (accented) used for the study were found to have a confusion rate (misidentified as the wrong syllable) of 29% when presented to native English speakers. The Japanese-accented productions could be perceived as either /r/ or /l/ by native English speakers on a proportion of the trials. The native English speakers were more accurate at identifying the unaccented English speech stimuli than the Japanese-accented speech stimuli. In contrast, the native Japanese speakers had difficulty identifying both the English-unaccented speech stimuli and the Japanese-accented stimuli. The following contrasts were investigated: (1) The neural processes that are involved in the perception of foreign-accented productions of a first language phonetic category were investigated using the contrast Eng(accented – unaccented) – Jpn(accented – unaccented). Subtracting the activity observed in the Jpn group controlled for general stimulus variables. (2) The contrast of Eng(accented) – Eng(unaccented) investigated which areas were involved in processing a difficult native phonetic identification task (accented) compared to those involved in processing an easy phonetic identification task (unaccented), without the potential confound of extraneous between group differences. However, acoustic stimulus characteristics were not controlled for by this contrast. (3) The neural processes selective for the perception of foreign-accented productions of a second language phonetic category, compared to foreign-accented productions of a first language phonetic category, were investigated using the contrast Jpn(accented) – Eng(accented). This contrast controlled for the neural processes that were related to task difficulty, such as attention and verbal rehearsal. (4) To investigate the overall neural processes involved in the perception of (native) unaccented productions of a second language phonetic category relative to the perception of unaccented productions of a first language phonetic category, we used the contrast Jpn(unaccented) – Eng(unaccented). This contrast did not control for task difficulty. All three of the contrasts above controlled for general processes related to performing a categorical perceptual identification task using a button response, though only the Jpn(accented) – Eng(accented) contrast additionally controlled for task difficulty.

A number of brain regions have been shown to be involved with the perception of unaccented/native productions of a second language phonetic category (Callan et al., 2003a, 2004a, 2006a; Wang et al., 2003) as well as foreign-accented speech (Adank et al., 2013). These regions include, but are not limited to: the ventral inferior premotor cortex including Broca's area (PMvi), the ventral superior and dorsal premotor cortex (PMvs/PMd), the superior temporal gyrus/sulcus (STG/S), and the cerebellum. If the neural processes involved in processing difficult-to-perceive speech sounds are dependent on the relative contribution of regions involved in articulatory planning control, then one might predict that the brain regions involved with speech motor control (PMvi/Broca's, PMvs/PMd, and the cerebellum) would be more active than regions involved with auditory processing (STG/S) when general acoustic differences in the stimuli are controlled.

As previously mentioned, the brain regions involved with internally simulating speech production (internal models) are hypothesized to constrain and facilitate speech perception, especially under degraded conditions (e.g., speech in noise, non-native speech) (Callan et al., 2003b, 2004a; Iacoboni and Wilson, 2006; Wilson and Iacoboni, 2006; Skipper et al., 2007; Iacoboni, 2008; Rauschecker and Scott, 2009; Rauschecker, 2011; Callan et al., 2014). Internal models are thought to simulate the input/output characteristics, or their inverses, of the motor control system (Kawato, 1999). With regards to speech production, inverse internal models predict the motor commands necessary to articulate a desired auditory (and/or orosensory) target (auditory-to-articulatory mapping). Forward internal models, conversely, predict the auditory (and/or orosensory) consequences of simulated speech articulation (articulatory-to-auditory mapping). It has been proposed that both forward and inverse internal models constrain and facilitate speech perception, especially under degraded conditions (Callan et al., 2004a, 2014; Rauschecker and Scott, 2009; Rauschecker, 2011). Facilitation is achieved by a process akin to analysis-by-synthesis (Stevens, 2002; Poeppel et al., 2008) (forward internal models: articulatory-to-auditory prediction) and synthesis-by-analysis (inverse internal models: auditory-to-articulatory prediction), specifically by competitive selection of the speech unit (phoneme, syllable, etc.) that best matches the ongoing auditory signal (or visual signal, in the case of audiovisual or visual-only speech). Brain regions thought to be involved with instantiating these articulatory-to-auditory and auditory-to-articulatory internal models include speech motor areas such as the PMC and Broca's area, the posterior regions of the STG/S, the IPL, and the cerebellum. In particular, the cerebellum, has been shown to instantiate internal models for motor control (Kawato, 1999; Imamizu et al., 2000), and there is evidence that it instantiates internal models related to speech (Callan et al., 2004a, 2007; Rauschecker, 2011; Tourville and Guenther, 2011; Callan and Manto, 2013). Brain activity in these regions (including the PMC, Broca's area, the IPL, and the cerebellum) during speech perception tasks has been used as evidence to support the involvement of motor processes during speech perception.

One potential criticism of ascribing activity found in speech motor regions to speech perception is that many of these same regions are known to be more active as a function of task difficulty. Activity in brain regions such as the IFG, the PMC, and the cerebellum has been shown to increase with task-related attentional demands and working memory (including verbal rehearsal) (Jonides et al., 1998; Davachi et al., 2001; Sato et al., 2009; Alho et al., 2012). As has been previously suggested (Hickok and Poeppel, 2007; Poeppel et al., 2008; Lotto et al., 2009; Scott et al., 2009), activity in these speech motor regions may not be related to speech perception intelligibility, but rather to other processes related to task difficulty. If these brain regions involved with speech motor processing are increasingly more active as a function of task difficulty, one would predict that subjects with worse phonetic identification performance (greater task difficulty) would show increased activity in these regions compared to subjects with better phonetic identification performance. However, the opposite result has been found, with an increase in PMC, IFG, and cerebellum activity associated with better phonetic identification performance on a difficult non-native phonetic category (Callan et al., 2004a). Similarly, PMC activity has been shown to be more active for correct compared to incorrect trials during a phonetic identification in noise task (Callan et al., 2010).

It is hypothesized that the perception of foreign-accented first language phonetic categories depends on the brain regions that instantiate the auditory—articulatory representation of phonetic categories. Research suggests that these regions include left hemisphere Broca's area and the PMC. In the case of the perception of second-language phonetic categories—for which the distinct second-language phonemes are subsumed within a single phonetic category in the native language (e.g., English /r/ and /l/ for native Japanese speakers)—additional neural processes may be recruited to establish new phonetic categories without interfering with the established native phonetic category. It is hypothesized that the establishment of these second-language phonetic categories (when the second-language is acquired after childhood) involves greater reliance on general articulatory-to-auditory feedback control systems, which generate auditory predictions based on articulatory planning, and are thought to be instantiated in right hemisphere PMC (Tourville and Guenther, 2011; Guenther and Vladusich, 2012).

## Methods

### Subjects

Thirteen right-handed native Japanese (Jpn) speakers with some English experience (at least 6 years of classes in junior and senior high school) and thirteen right-handed native English (Eng) speakers participated in this study. The native Japanese-speaking subjects were nine females and four males whose ages ranged from 23 to 37 years (*M* = 30.4 years, *SD* = 4.5). The native English-speaking subjects were one female and twelve males whose ages ranged from 21 to 39 years (*M* = 27.8 years, *SD* = 5.1). All subjects included in this study scored significantly above chance when they identified the /r/ and /l/ productions of a native English speaker, which ensured that all subjects were actively trying to do the task. Subjects were paid for their participation, and gave written informed consent for the experimental procedures, which were approved by the ATR Human Subject Review Committee in accordance with the principles expressed in the Declaration of Helsinki.

### Stimuli and procedure

The stimuli were acquired from the speech database compiled by the Department of Multilingual Learning (ATR—HIS, Kyoto, Japan). The experiment had two, within-subject conditions: a foreign-accented speech condition and an unaccented speech condition. These two conditions were composed of audio speech stimuli consisting of English syllables beginning with a /r/ or /l/, which were followed by five different following English vowel contexts (/a, e, i, o, u/). There were three occurrences of each syllable for each accent condition for a total of 60 trials in the experiment. All stimuli were recorded digitally in an anechoic chamber with a sampling rate of 44,100 Hz. The unaccented speech was taken from samples of female and male native English speakers. The foreign-accented speech was taken from samples of female and male native Japanese speakers that produced /r/–/l/ confusions (*M* = 29%, *SD* = 13%), as determined by a forced-choice identification task performed by native English speakers (the number of evaluators ranged from 6 to 10 individuals, depending on the stimulus). Both the foreign-accented and unaccented /r/ and /l/ stimuli consisted of six female voices and nine male voices. The stimuli were down-sampled to 22,050 Hz for presentation during the experiment.

The fMRI procedure consisted of an event-related design in which the sequence of presentation of the various stimulus conditions (unaccented /r/, unaccented /l/, foreign-accented /r/, foreign-accented /l/, and /null trial/) was generated stochastically using SPM99 (Wellcome Department of Cognitive Neurology, UCL). An event-related design was employed so that the various stimulus conditions could be presented (approximately 85–90 dB SPL) in a pseudo-random order. This ensured that subjects could not predict which stimulus would occur during the subsequent presentation. Stimuli were presented (synchronized with fMRI scanning using Neurobehavioral System's Presentation software) via MR-compatible headphones (Hitachi Advanced Systems' ceramic transducer headphones; frequency range 30–40,000 Hz, approximately 20 dB SPL passive attenuation). Subjects identified whether the stimuli started with /r/ or /l/, and indicated which they perceived by pressing a button with their left thumb. The left hand was used instead of the right hand so that brain activity in left Broca's area and left PMC could be better identified, with less influence of activity associated with the button-press motor response. The identity of the buttons was counterbalanced across subjects. Stimuli were presented at a rate of approximately 2250 ms in a pseudo-random order dependent on the event sequence. Subjects were asked to respond quickly to minimize differences in the hemodynamic response resulting from long response times (Poldrack, 2000). However, they were not asked to respond as quickly as they could, therefore response latencies were not evaluated. Null trials in which only silence occurred were also included and used as a baseline condition. Subjects were not given online feedback regarding the correctness of their responses. All subjects were given a practice session outside of the scanner using stimuli similar to those used in the experimental session.

Each subject participated in multiple experiments, including the present study, within the same insertion into the fMRI scanner. The order of the different experiments was counterbalanced across subjects. Depending on the number of experiments in which a subject participated, the total time in the scanner ranged from approximately 30–60 min. The session lasted approximately 7 min for this experiment.

### fMRI data collection and preprocessing

For functional brain imaging, Shimadzu-Marconi's Magnex Eclipse 1.5T PD250 was used at the ATR Brain Activity Imaging Center. Functional T2^*^ weighted images were acquired using a gradient echo-planar imaging sequence (echo time 55 ms; repetition time 2000 ms; flip angle 90°). A total of 20 contiguous axial slices were acquired with a 3 × 3 × 6 mm voxel resolution covering the cortex and cerebellum. For some subjects, 20 slices was not a sufficient number to cover the entire cortex and thus the top part of the cortex was missing. As a result, the analyses conducted in this study do not include the top part of the cortex. A total of 304 scans were taken during a single session. Images were preprocessed using programs within SPM8 (Wellcome Department of Cognitive Neurology, UCL). Differences in acquisition time between slices were accounted for; images were realigned and spatially normalized to a standard space using a template EPI image (3 × 3 × 3 mm voxels), and were smoothed using a 6 × 6 × 12 mm FWHM Gaussian kernel.

### Statistical image analysis

Regional brain activity for the various conditions was assessed with a general linear model using an event-related design. Realignment parameters were used to regress out movement-related artifacts. In addition, low-pass filtering, which used the hemodynamic response function, was employed. The event-related stochastic design used to model the data included null responses and a stationary trial occurrence probability. A mixed-effects model was employed. A fixed-effect analysis was first employed for all contrasts of interest across data from each subject separately. The contrasts of interest for both the Jpn and Eng subjects included: unaccented speech relative to baseline; accented speech relative to baseline; and accented relative to unaccented speech. At the random effects level between subjects, the contrast image of the parameter estimates of the first level analysis for each subject was used as input for a SPM model employing two-sample *t*-tests. The contrasts of interest consisted of the following: (1) Processes related to the perception of first language phonetic contrasts in accented speech Eng(accented – unaccented) – Jpn(accented – unaccented); (2) Processes related to the perception of first language accented speech (difficult task) relative to first language unaccented speech (easy task). (3) Processes related to the perception of foreign-accented speech Jpn(accented) – Eng(accented) and (4) Processes related to the perception of unaccented productions of a second language phonetic category Jpn(unaccented) – Eng(unaccented). Because the study is quasi-experimental in the sense that assignment of participant into Eng and Jpn groups is not random, the variance not attributable to the independent experimental variables (e.g., educational experience and cultural differences related to carrying out the tasks) may significantly influence participants' performance and neural responses, which could potentially confound the results. To ensure that the differential brain activity related to the contrasts of interest (given above) were not the result extraneous neural processes involved with behavioral performance, task difficulty, and/or variables arising from the quasi-experimental design, the random-effects analyses were conducted using the raw percent correct phonetic identification performance scores as a covariate of non-interest.

A False Discovery Rate (FDR) correction for multiple comparisons across the entire volume was employed with a threshold of pFDR < 0.05 using a spatial extent greater than 5 voxels. If no voxels were found to be significant using the FDR, a correction threshold of *p* < 0.001 uncorrected with a spatial extent threshold greater than 5 voxels was used. Region of interest (ROI) analyses were conducted using MNI coordinates for the PMvi/IFG (left −51,9,21; right 51,15,18), the PMvs (left −36,−3,57; right 27,−3,51), the STG/S (left −57,−39,9) and the cerebellum (left −27,−63,−39; right 30,−66,−33) given that in Callan et al. (2004a) these regions were found to be involved in processing difficult-to-perceive speech contrasts. It should be noted that these coordinates (for PMvi/IFG and STG/S) fall within the cluster of activity in regions found to be active for perception of accented speech, as reported by Adank et al. (2013). Small volume correction for multiple comparisons was carried out using the seed voxels reported above within a sphere with a radius of 8 mm. The location of active voxels was determined by reference to the Talairach atlas (Talairach and Tournoux, 1988) as well as by using the Anatomy Toolbox within SPM8. Activity in the cerebellum was localized with reference to the atlas given by Schmahmann et al. (2000).

## Results

### Behavioral performance

**Figure 1:**
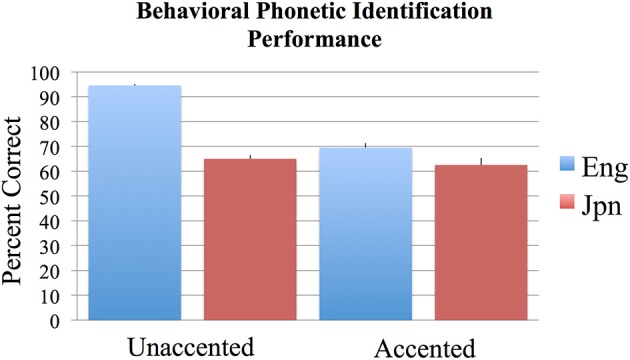
**Mean percent correct behavioral phonetic (/r/ vs. /l/) identification performance for the English (blue) and Japanese (red) groups for unaccented and foreign-accented speech**. Standard error of the mean is given above each bar. All conditions were significantly above chance performance of 50%. See text for additional contrasts that were statistically significant.

The results of the two-alternative forced-choice phoneme identification task (in percent correct) were analyzed across subjects using an ANOVA with the two factors of language group (Jpn and Eng) and accent (unaccented and accented). Bonferroni corrections for multiple comparisons were used to determine statistical significance at *p* < 0.05 for all behavioral analyses conducted. The results are as follows: the interaction between Jpn and Eng subjects for accented and unaccented stimuli was significant [Eng unaccented: *M* = 94.6%, *SE* = 0.6; Jpn unaccented: *M* = 69.5%, *SE* = 1.8; Eng accented: *M* = 65.0%, *SE* = 1.5; Jpn accented: *M* = 62.5%, *SE* = 2.8; *F*_(1, 48)_ = 40.2, *p* < 0.05 corrected] (see Figure 1). The main effect of group (Eng > Jpn) was significant [Eng *M* = 79.8%, *SE* = 3.11, Jpn *M* = 66.0%, *SE* = 1.8, *F*_(1, 48)_ = 60.3, *p* < 0.05 corrected]. The main effect of accent (unaccented > accented) was also significant [unaccented: *M* = 82.1%, *SE* = 2.9, accented: *M* = 63.7%, *SE* = 1.1, *F*_(1, 48)_ = 106.4, *p* < 0.05 corrected]. The identification performance on the two-alternative forced-choice task was significantly greater than chance for the unaccented and accented conditions for both Eng and Jpn subjects (see Figure 1) [Jpn unaccented: *T*_(12)_ = 7.2, *p* < 0.05 corrected; Jpn accented: *T*_(12)_ = 7.2, *p* < 0.05 corrected; Eng unaccented: *T*_(12)_ = 75.1, *p* < 0.05 corrected; Eng accented: *T*_(12)_ = 10.7, *p* < 0.05 corrected]. The Eng subjects had significantly better performance than the Jpn subjects for the unaccented speech stimuli condition [*T*_(12)_ = 9.4; *p* < 0.05 corrected]. For accented stimuli, there was no significant difference for identification (evaluated based on the intended production of the stimuli) between native English speaking subjects and native Japanese speaking subjects [*T*_(24)_ = 1.1; *p* = 0.27 uncorrected]. There was also no significant difference between Eng subjects' performance for the accented stimuli and Jpn subjects' performance for the unaccented stimuli [*T*_(24)_ = 1.13, *p* = 0.15 uncorrected]. For Eng subjects there was a significant difference between performance for the unaccented and accented stimuli [*T*_(12)_ = 18.2, *p* < 0.05 corrected]. The difference for Jpn subjects between the performance for unaccented and accented stimuli was not significant when corrections were made for multiple comparisons, but the difference was significant using an uncorrected threshold [*T*_(12)_ = 3.3, *p* < 0.01 uncorrected].

### Brain imaging

**Figure 2:**
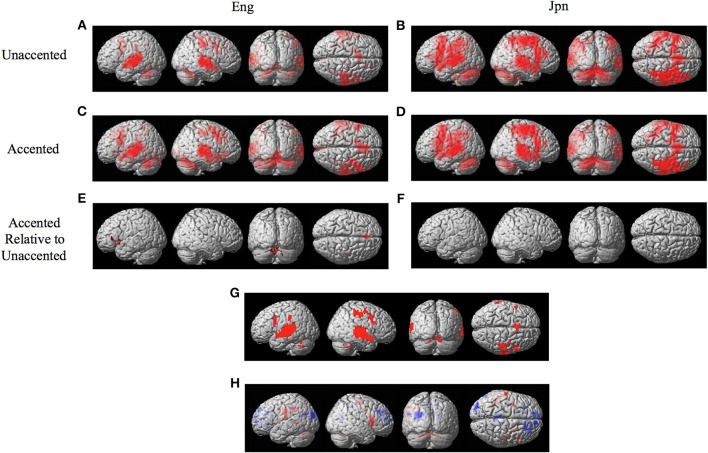
**Significant brain activity (thresholded at pFDR < 0.05 corrected) for the contrast of (A) Eng (unaccented), (B) Jpn (unaccented), (C) Eng (accented), and (D) Jpn (accented)**. All contrasts showed activity bilaterally in premotor cortex and Broca's area, the superior temporal gyrus/sulcus, the inferior parietal lobule, the pre- supplementary motor area pre-SMA, and the cerebellum. The conjunction analysis, shown in **(G)**, confirmed these regions were active for all conditions **(E)**. The contrast of accented Relative to unaccented thresholded at *p* < 0.001 uncorrected for Jpn showed activity in the left inferior frontal gyrus in Broca's area 44, the right dorsal premotor cortex, the pre-SMA, and the cerebellum bilaterally **(F)**. The contrast of accented relative to unaccented for the Jpn group did not show any significant activity thresholded at *p* < 0.001 uncorrected. The main effect of language group (Japanese vs. English) is shown in **(H)**, red corresponds to activity thresholded at *p* < 0.001 for Japanese > English and blue corresponds to activity for English > Japanese.

The random effects one-sample *t*-test of the unaccented and accented condition relative to the null condition (background scanner noise) was carried out separately for Jpn and Eng groups. A FDR correction for multiple comparisons across the entire volume was used with a threshold of pFDR < 0.05 (spatial extent > 5 voxels). The results for unaccented and accented conditions for both the Eng and Jpn groups (see Figures 2A–D) indicated extensive activity in regions of the brain known to be involved with speech processing bilaterally (STG/S, including primary auditory cortex, MTG, SMG, Broca's area, PMC, medial frontal cortex MFC/pre-suplementary motor area pre-SMA, anterior cingulate cortex ACC, cerebellar lobule VI, cerebellar Crus I). Activity associated with the motor response of pushing the button with the left thumb was also present for both the Jpn and Eng groups in the right motor and somatosensory cortex. The conjunction analysis, which determined the intersection of active voxels for all conditions thresholded at pFDR < 0.05, showed activity in most of the above-mentioned regions (see Figure 2G and Table 1).

**Table 1:** **Conjunction of all conditions Eng Unaccented, Jpn Unaccented, Eng Accented, Jpn Accented (Figure 2G)**.

**Brain region**	**MNI coordinates**
PMvi, Broca's area, BA 6,44	−54,12,27
	51,6,21
PMvs/PMd BA 6	−51,6,39
	54,9,39
	39,−12,51
PostCG, IPL BA1,2	54,−30,51
	−45,−30,39
Medial Frontal Cortex BA 9 Pre-SMA	−6,12,57
SPL BA7	−27,−57,45
Insula BA13	−36,−33,24
MTG/STG BA21,22	−63,−27,−3
	66,−27,−3
Cerebellum Vermis	0,−78,−18
Cerebellum Lobule VI	−18,−54,−24
	27,−66,−27

Table showing clusters of activity for the conjunction of all contrasts relative to rest (Eng Unaccented, Eng Unaccented, Jpn Accented, Jpn Unaccented) thresholded at pFDR < 0.05 corrected with an extent threshold greater than 5 voxels. Jpn., Japanese; Eng., English; Cor., corrected for multiple comparisons; BA, Brodmann area; PMvi, Ventral inferior premotor cortex; PMvs, Ventral superior premotor cortex; PMd, Dorsal premotor cortex; PostCG, Postcentral gyrus; IPL, Inferior parietal lobule; pre-SMA, Pre-supplementary motor area; SPL, Superior parietal lobule; MTG, Middle temporal gyrus; STG, Superior temporal gyrus. Negative × MNI coordinates denote left hemisphere and positive × values denote right hemisphere activity.

The interaction effect between the factors of language group and accent is discussed below. The main effect of accent (accented vs. unaccented) did not show any significant differential activity using a corrected threshold of pFDR < 0.05 or an uncorrected threshold of *p* < 0.001 (spatial extent > 5 voxels). The main effect of language group (Jpn vs. Eng, see Figure 2H and Table 2) showed significant differential activity for Japanese > English (red) *p* < 0.001 (spatial extent > 5 voxels), predominantly in left and right PMvi/Broca's area, PMvs/PMd, the postcentral gyrus, the cerebellum, and the left inferior parietal lobule. The significant differential activity for Eng > Jpn (blue) *p* < 0.001) (spatial extent > 5 voxels) was present predominantly in the medial frontal gyrus, the middle frontal gyrus, the anterior cingulate cortex, and the middle cingulate cortex.

**Table 2:** **Main contrast of language group**.

**Brain region**	**Jpn – Eng Accented + Unaccented Figure 2H (red)**	**Eng – Jpn Accented + Unaccented Figure 2H (blue)**
PMvi, Broca's area, BA 6,44	−45,0,8	
	48,12,9	
PMvs/PMd BA 6	−30,0,36	
	30,0,39	
	39,−15,60	
PostCG, IPL BA1,2	−60,−18,21	
	51,−24,60	
PostCG, IPL BA3		−30,−24,48
Superior medial gyrus BA10		−9,54,0
Medial frontal gyrus/SFG BA9		−30,30,24
		−15,51,39
		18,33,33
Middle frontal gyrus BA11		−30,34,−19
Anterior cingulate gyrus		9,51,15
Middle cingulate cortex BA24,31		−12,−39,42
		12,−33,45
		12,−3,45
IPL BA40	−45,−39,39	
SPL BA7		
Insula BA13, 47	36,18,−9	−36,−18,15
MTG /STG BA21,22	−51,−48,6	
Angular gyrus BA39		−54,−66,24
MOG BA18,19	−27,−69,30	−36,−87,27
Cuneus/Precuneus		−9,−72,24
		24,−63,18
Lingual gyrus BA18		−9,−60,0
Cerebellum	−27,−69,−30	
Lobule VIIa Crus I	−39,−69,−36	
	21,−66,−36	
Cerebellum	−6,−57,−30	
Lobule V		
Cerebellum	−15,−72,−27	
Lobule VI		
Putamen	30,9,0	

Table showing clusters of activity for the main effect of language group thresholded at pFDR < 0.05 corrected with an extent threshold greater than 5 voxels. Jpn., Japanese; Eng., English; Cor., corrected for multiple comparisons; BA, Brodmann area; PMvi, Ventral inferior premotor cortex; PMvs, Ventral superior premotor cortex; PMd, Dorsal premotor cortex; PostCG, Postcentral gyrus; SFG, Superior Frontal Gyrus; IPL, Inferior parietal lobule; SPL, Superior Parietal Lobule; MTG, Middle Temporal Lobe; STG, Superior Temporal Lobe; MOG, Middle Occipital Gyrus. Negative × MNI coordinates denote left hemisphere and positive × values denote right hemisphere activity.

The contrast of accented relative to unaccented speech was carried out separately for Eng and Jpn subjects. For both Eng and Jpn subjects, no significant activity was found using a corrected threshold of pFDR < 0.05; therefore, a threshold of *p* < 0.001 uncorrected was used. For Eng subjects, activity was found to be present in left PMvi/Broca's area, right PMvs/PMD, left Broca's area BA 45, left IFG BA 47, the pre-SMA, and left and right cerebellar lobules VI and VIIa (see Figure 2E and Table 3). The results of the region of interest analysis (ROI) using small volume correction for multiple comparisons revealed significant activity in the left and right cerebellum lobule VI, and a trend toward significant activity in the left PMvi/Broca's, the right PMvs/PMd, and the left STG/S (see Table 4). To ensure that the differential brain activity reported in the analyses of this study was not just the result extraneous neural processes involved with (or resulting from) behavioral performance, task difficulty (e.g., attention, working memory, concentration and/or response confidence), and/or variables arising from the quasi-experimental design, the same analyses were conducted using phonetic identification performance as a covariate of non-interest. The results of the contrast Eng(accented) – Eng(unaccented) using phonetic identification performance as a covariate of non interest showed activity in left PMvi/Broca's area, left Broca's BA 45, pre-SMA, right cerebellum Lobule VI, and left cerebellum lobule VII (see Table 3). The ROI analysis using phonetic identification performance as a covariate of non-interest revealed significant activity in left and right cerebellum lobule VI, and a trend toward significant activity in left PMvs (*p* < 0.057) (see Table 4). No significant activity was found for Jpn subjects using a threshold of *p* < 0.001 uncorrected or for the ROI analyses (see Figure 2F and Tables 3, 4).

**Table 3:** **MNI Coordinates of Clusters of Activity for Contrasts of Interest**.

**Brain region**	**Accented – Unaccented/rl/ Identification (Eng – Jpn) Figure 3**	**Accented – Unaccented/rl/ Identification(Eng) Figure 2E**	**Accented/rl/ Identification (Jpn – Eng) Figure 5A**	**Unaccented/rl/ Identification (Jpn – Eng) Figure 5B**
PMvi, Broca's area, BA 6,44		−48,10,4 (−48,12,−3)	(−45,0,9)	(48,3,0) 48,9,15, 60,15,3
			48,12,6 (48,12,6)	
PMvs/PMd BA 6		33,−15,48	27,0,36 (27,0,36)	30,0,42 (30,0,42)
				(57,0,45) (39,−15,66)
Broca's Area BA 45	−51,30,6 (−54,30,3^*^) 54,27,18	−42,33,6 (−48,30,9)		54,21,9
IFG BA47		−45,24,−12 (−45,24,−12)		−30,21,−4
Rolandic operculum				−63,−18,21 (57,−12,12)
BA43				
MFG BA8		(−51,15,42)		
MFC including	0,39,33^**^, 0,36,42	0,32,38, 0,29,50 (3,33,45)		
Pre-SMA				
SMA				(−15,−6,66)
DLPFC	54,30,30			
MTG BA21				(69,−18,−6)
IPL BA 40				−45,−39,39, −30,−48,39
SPL				(−12,−51,66)
Cerebellum Lobule V				−15,−57,−30
Cerebellum Lobule VI		27,−60,−33 (27,−60,−33)	(−15,−57,−27)	21,−66,−36
				(−18,−57,−30)
Cerebellum Lobule VII	6,−81,−33	(−3,−69,−30)		−27,−69,−30
		−9,−87,−27		
		18,−72,−39		
Brain Stem	0,−30,−30			(6,−45,−36)

Table showing clusters of activity for the various contrasts thresholded at p < 0.001 uncorrected. Coordinates in Parentheses denote those that are significant when using phonetic identification performance as a covariate of non-interest. Jpn., Japanese; Eng., English; BA, Brodmann area; PMvi, Ventral inferior premotor cortex; PMvs, Ventral superior premotor cortex; PMd, Dorsal premotor cortex; IFG, Inferior frontal gyrus; MFG, Middle frontal gyrus; MFC, Medial frontal cortex. SMA, Supplementary motor area; DLPFC, Dorsolateral Prefrontal Cortex; MTG, Middle Temporal Gyrus; IPL, Inferior Parietal Lobule; SPL, Superior parietal lobule. Negative × MNI coordinates denote left hemisphere and positive × values denote right hemisphere activity.

* Cluster was not significant when thresholded at p < 0.001 uncorrected but was significant at p < 0.0015 uncorrected.

** Significant at p < 0.05 FWE correcting for multiple comparisons across the entire volume.

**Table 4:** **ROI analysis using small volume correction for contrasts of interest**.

**Brain region**	**SVC center Coordinate (8 mm radius)**	**Accented – Unaccented /rl/ identification (Eng –Jpn) Figure 4**		**Accented – Unaccented /rl/ Identification (Eng) Figure 2E**		**Accented /rl/ Identification (Jpn –Eng) Figure 6**		**Unaccented /rl/ Identification (Jpn –Eng) Figure 7**	
		**pCor**.	**x,y,z**	**pCor**.	**x,y,z**	**pCor**.	**x,y,z**	**pCor**.	**x,y,z**
PMvi, Broca's BA6,44	−51,9,21	0.030	−48,12,21	0.092	−57,6,21	n.s.	–	0.042	−54,9,27
	51,15,18	n.s.	–	n.s.	–	0.045	48,9,15	0.006	48,9,15
	**CovPerf**								
	−51,9,21	n.s.	–	n.s.	–	n.s.	–	n.s.	–
	51,15,18	n.s.	–	n.s.	–	0.074	48,12,12	n.s.	–
PMvs	−36,−3,57	n.s.	–	0.081	−36,0,51	n.s.	–	n.s.	–
	27,−3,51	n.s.	–	n.s.	–	0.036	27,0,45	0.006	27,0,45
	**CovPerf**								
	−36,−3,57	n.s.	–	0.057	−36,0,51	n.s.	–	n.s.	–
	27,−3,51	n.s.	–		–	0.063	27,0,45	0.027	21,−6,51
STG/S	−57,−39,9	n.s.	–	0.075	−57,−36,3	n.s.	–	n.s.	–
	**CovPerf**								
	−57,−39,9	n.s.	–	0.091	−57,−36,3	n.s.	–	n.s.	n.s.
Cerebellum Lobule VI	−27,−63,−39	n.s.	–	0.011	−30,−57,−36	0.042	−27,−66,−33	0.011	−27,−66,−33
	30,−66,−33	0.034	36,−72,−33	0.025	27,−60,−33	n.s.	–	0.028	24,−69,−33
	**CovPerf**								
	−27,−63,−39	n.s.	–	0.012	−30,−57,−36	0.039	−27,−66, −33	0.005	−21,−60,−39
	30,−66,−33	n.s.	–	0.024	27,−60,−33	n.s.	–	0.033	33,−63,−39

Table showing results of small volume correction analysis (p < 0.05) for multiple comparisons for selected contrasts within regions of interest using MNI coordinates specified in Callan et al. (2004a) as the seed voxels. The first set of results is for the original analysis. The second set of results, under the heading of CovPerf, is for the analysis in which phonetic identification performance is used as a covariate of non-interest. SVC, Small volume correction; ROI, Region of Interest; BA, Brodmann area; PMvi, Premotor cortex ventral inferior; PMvs, Premotor cortex ventral superior. n.s., Not significant at p < 0.05 corrected. pCor., p corrected for multiple comparisons within the SVC small volume corrected region of interest. Negative × MNI coordinates denote left hemisphere and positive × values denote right hemisphere activity.

**Figure 3:**
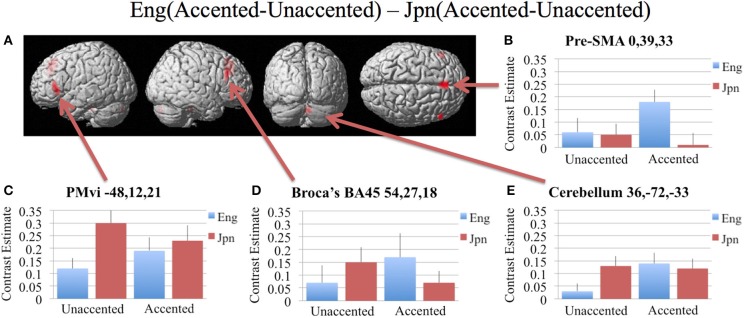
**Significant brain activity (thresholded at *p* < 0.001 uncorrected) for the interaction of language group and accent**. This contrast focused on the activity involved with perception of foreign-accented productions of a first-language phonetic category. **(A)** Significant brain activity rendered on the surface of the brain for the contrast of Eng(accented-unaccented) – Jpn(accented-unaccented) showing activity in pre- supplementary motor area pre-SMA, left and right Broca's area BA45, right dorsolateral prefrontal cortex DLPFC, and left and right cerebellum. **(B–E)** shows contrast estimates and standard error of the SPM analysis relative to rest for the four conditions in selected regions: **(B)** Pre-SMA, **(C)** PMvi, **(D)** Broca's, **(E)** Cerebellum.

**Figure 4:**
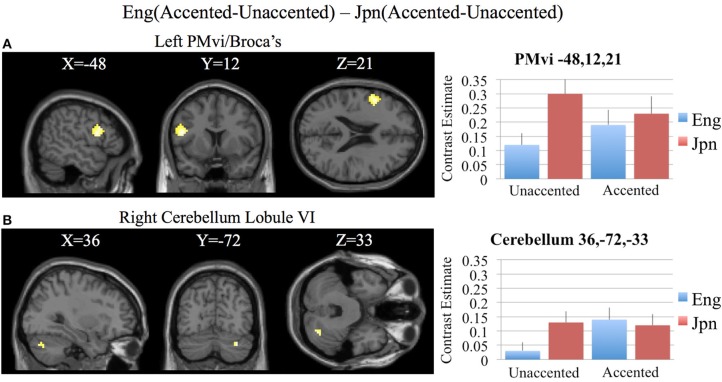
**Region of interest (ROI) analysis for the contrast of Eng(accented-unaccented) – Jpn(accented-unaccented) using small volume correction (*p* < 0.05) for multiple comparisons. (A)** left PMvi/Broca's area. **(B)** Right Cerebellum Lobule VI. MNI X, Y, Z coordinates are given at the top of each brain slice. Negative × MNI coordinates denote left hemisphere and positive × values denote right hemisphere activity. The SPM contrast estimates and standard error relative to rest for all four conditions are given on the left side of each ROI rendered image.

In order to determine brain activity that was related to difficult perceptual identification of a native phonetic contrast, the foreign-accented condition (which was difficult to perceive for both the native English speakers and the native Japanese speakers) was compared to the unaccented condition (which was easy to perceive for the native English speakers, but more difficult to perceive for the native Japanese speakers) between the Eng vs. the Jpn group using the contrast Eng(accented – unaccented) – Jpn(accented – unaccented) (random effects two-sample *t*-test). Only the pre-SMA activity was significant at *p* < 0.05 FDR corrected, therefore the analysis was conducted using a threshold of *p* < 0.001 uncorrected. Brain regions that showed significant differential activity for this contrast included the left and right Broca's area BA45, the pre-SMA, the right dorsolateral prefrontal cortex (DLPFC), the cerebellum lobule VIIa, and the brain stem (see Figure 3 and Table 3). The same analysis using phonetic identification performance as a covariate of non-interest revealed activity only in left Broca's area using a threshold of *p* < 0.0015. The results of the ROI analysis using small volume correction for multiple comparisons revealed significant activity in the left PMvi, and the right cerebellum lobule VI (see Figure 4 and Table 4). When using performance as a covariate of non-interest, no significant differential activity was found when correcting for multiple comparisons within the ROIs (Table 4).

**Figure 5:**
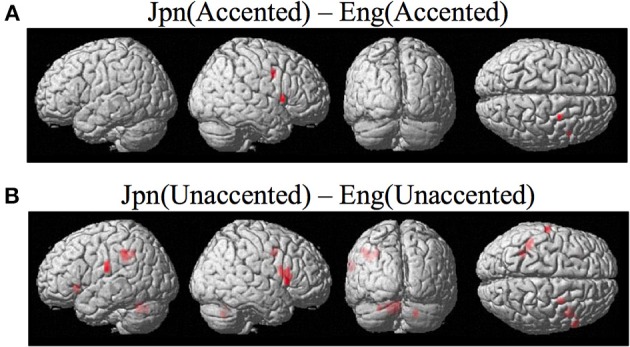
**(A)** Contrast investigating specific brain regions involved with the perception of foreign-accented productions of a second-language phonetic category, Jpn(accented) – Eng(accented). Activity is present in the right ventral inferior premotor cortex including Broca's area PMvi/Broca's right ventral superior premotor cortex PMvs. **(B)** Activity for perception of foreign-accented productions of a second language phonetic category that may not be specific Jpn(unaccented) – Eng(unaccented) was found in the left and right PMvi/Broca's, the right PMvs/PMd, the right Broca's area BA45, the left inferior frontal gyrus BA47, the left postcentral gyrus, the left inferior parietal lobule, and the left and right cerebellum.

**Figure 6:**
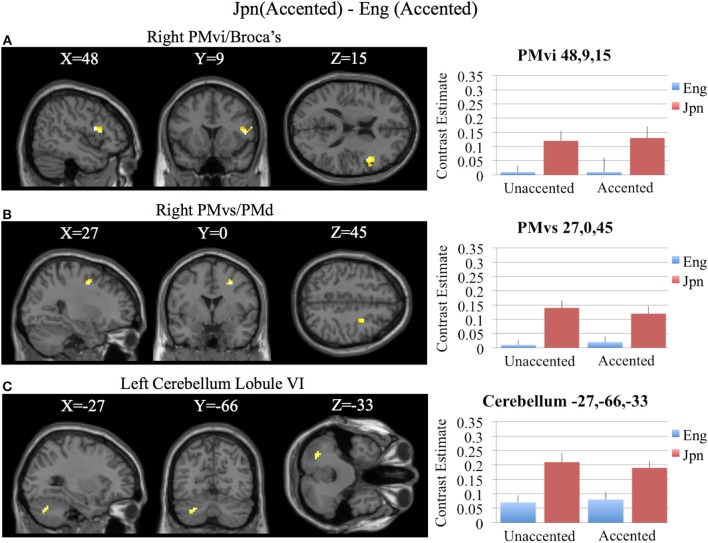
**Region of interest analysis for the contrast of Jpn(accented) – Eng(accented) using small volume correction (*p* < 0.05) for multiple comparisons. (A)** Right PMvi/Broca's area. **(B)** Right PMvs/PMd. **(C)** Left Cerebellum Lobule VI. MNI X, Y, Z coordinates are given at the top of each brain slice. Negative × MNI coordinates denote left hemisphere and positive × values denote right hemisphere activity. The SPM contrast estimates and standard error relative to rest for all four conditions are given on the left side of each ROI rendered image.

Brain activity related to processing of foreign-accented productions of a second language phonetic category that was different from processing of foreign-accented productions of a first language phonetic category was investigated using the contrast Jpn(accented) – Eng(accented). No significant activity was found using a corrected threshold of pFDR <0.05, therefore a threshold of *p* < 0.001 uncorrected was used. Activity was present in the right PMvi/Broca's area and the right PMvs/PMd. Using phonetic identification performance as a covariate of non-interest revealed activity in right PMvi/Broca's area and right PMvs/PMd (see Figure 5A and Table 3). Using phonetic identification performance as a covariate of non-interest revealed activity in right PMvi/Broca's area, the right PMvs/PMd, and the left cerebellar lobule VI. For the ROI analysis, activity was significant in the right PMvi/Broca's area, right PMvs/PMd, and the left cerebellar lobule VI (see Figure 6 and Table 4). Using performance as a covariate of non-interest, the ROI analysis showed significant activity in left cerebellar lobule VI, and a trend toward significance in both right PMvi/Broca's area (*p* < 0.074) and right PMvs/PMd (*p* < 0.063) (see Table 4).

**Figure 7:**
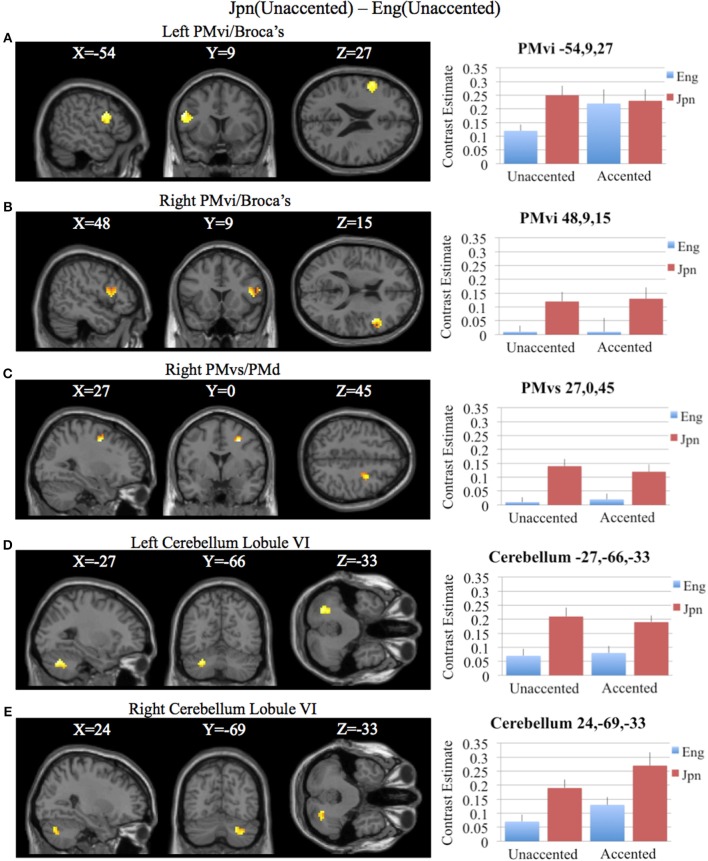
**Region of interest analysis for the contrast of Jpn(unaccented) – Eng(unaccented) using small volume correction (*p* < 0.05) for multiple comparisons. (A)** Left PMvi/Broca's area. **(B)** Right PMvi/Broca's area. **(C)** Right PMvs/PMd. **(D)** Left Cerebellum Lobule VI. **(E)** Right Cerebellum Lobule VI. MNI X, Y, Z coordinates are given at the top of each brain slice. Negative × MNI coordinates denote left hemisphere and positive × values denote right hemisphere activity. The SPM contrast estimates and standard error relative to rest for all four conditions are given on the left side of each ROI rendered image.

To determine activity related to processing of unaccented productions of a second language phonetic category that was different from that of unaccented productions of a first language phonetic category, the difference between the Jpn and Eng subjects for unaccented speech was investigated using the contrast Jpn(unaccented) – Eng(unaccented). No significant activity was found using a corrected threshold of pFDR < 0.05, therefore, a threshold of *p* < 0.001 uncorrected was used. Activity was present in left and right PMvi/Broca's area, right PMvs/PMd, right Boca's BA45, left IFG BA47, left PostCG, left IPL, and left cerebellar lobules VIIa and V, as well as left and right cerebellar lobule VI (see Figure 5B and Table 3). Using phonetic identification performance as a covariate of non-interest, the analysis revealed activity primarily in right PMvi/Broca's area, right PMvs/PMd, and left cerebellum lobule VI. The results of the ROI analysis using small volume correction for multiple comparisons revealed significant activity in left and right PMvi/Broca's, right PMvs/PMd and left and right cerebellum lobule VI (see Figure 7 and Table 4). These same brain regions were shown to have significant activation (correcting for multiple comparisons) when using phonetic identification performance as a covariate of non-interest.

## Discussion

The goal of this study was to determine if there are differences in the level and/or patterns of activation for various brain regions involved with the processing of accented speech when distinct phonetic categories existed within a listener's language networks (first-language), relative to when listeners do not have well established phonetic categories (second-language) (i.e., English /r/ and /l/ identification for native Jpn speakers). The conjunction analysis of all four conditions [Eng(accented), Eng(unaccented), Jpn(accented), Jpn(unaccented)] revealed that the same brain regions (STG/S, MTG, SMG, Broca's area, PMC, medial frontal cortex MFC/pre-suplementary motor area, and the cerebellum lobule VI) were active (see Figures 2A–D,G and Table 1). These results suggest that, to a large extent, it is the *level* of activity within these common regions that differs between conditions, rather than recruitment of different regions in the brain. It should be noted that, even for the Eng unaccented condition, there was common activation in speech motor regions.

Increased brain activity during the presentation of accented first-language phonetic categories relative to unaccented phonetic categories [Eng(accented – unaccented)] was located primarily in the left and right cerebellum, as well as in left PMvi/Broca's area, and right PMvs/PMd (see Figure 2E, Tables 3, 4). These results were also found when using phonetic identification performance as a covariate of non-interest. When general stimulus and subject variables were controlled for, using the contrast of Eng(accented – unaccented) – Jpn(accented – unaccented), the brain regions with significant activation included the pre-SMA, the right cerebellum, left Broca's area BA45, and the left PMvi/Broca's area (see Figures 3, 4, Tables 3, 4). However, when using performance as a covariate of non-interest, only left Broca's area BA45 showed significant activity (see Tables 3, 4). Broca's area BA45 is thought to provide a contextual supporting role to the mirror neuron system (Arbib, 2010). PMvi/Broca's area and the cerebellum are hypothesized to be regions that instantiate the articulatory—auditory models that are involved with both speech production and perception (Callan et al., 2004a; Tourville and Guenther, 2011; Guenther and Vladusich, 2012). The left hemisphere activity observed in Broca's area BA 45 and PMvi/Broca's area, is consistent with other studies that showed only left hemisphere activity for speech perception tasks that required phonetic processing (Demonet et al., 1992; Price et al., 1996). The presence of increased activity in speech motor regions observed in this study, and the lack of significant differential activity in the STG/S, are consistent with the hypothesis that neural processes involved with auditory—articulatory mappings are used to facilitate the perception of foreign-accented productions of one's first language. However, the absence of differential activity in auditory regions for this contrast does not indicate that auditory processes are not important for intelligibility and perceptual categorization.

The activity present in the MFC that included the pre-SMA for all conditions (see Figure 2 and Table 1) is interesting given that several studies suggest that this region may be involved with value and contex-dependent selection of actions (Deiber et al., 1999; Lau et al., 2004; Rushworth et al., 2004). Activity found in the MFC/Pre-SMA in this study may represent value and context dependent selection of internal models. It is important to note that the contrast Eng (accented) vs. Jpn (accented) showed greater activity in the MFC (see Figures 3, 4, Tables 3, 4). This was also true when phonetic identification performance was used as a covariate of non-interest. This suggests greater use of value-dependent context for selection when internal models are well established (as is thought to be the case for /r/ and /l/ for native English speakers). This region was also displayed significant activation when the Eng vs. Jpn groups were compared (see Figure 2H, Table 2). The greater extent of activity in these regions compared to the Callan et al. (2004a) study may be explained by the larger number of speakers used for the stimuli in this study, which could have resulted in considerably more context variability.

Brain regions specific to the perception of foreign-accented productions of phonetic categories from one's second language, when controlling for task difficulty [Jpn(accented) – Eng(accented)], was localized in right PMvi/Broca's area, right PMvs/PMd, and the left cerebellum (see Figures 5A, 6, and Tables 3, 4). These results are also true when using phonetic identification performance as a covariate of non-interest. Task difficulty was controlled for by presenting foreign accented speech (English /rl/ phonetic contrast) that was difficult for both native English and native Japanese speakers to correctly identify. It is important to point out that behavioral performance during the fMRI experiment revealed no significant difference between native English and native Japanese speakers for the foreign accented stimuli, which suggests similar levels of task difficulty for both groups.

The contrast Jpn(unaccented) – Eng(unaccented) revealed activity in right PMvi/Broca's, right PMvs/PMd, the right and the left cerebellum (see Figures 5B, 7 and Tables 3, 4). Activity in these regions was also present when using phonetic identification as a covariate of non-interest. The presence of activity in right PMvs/PMd for the Jpn(accented) – Eng(accented) contrast and the Jpn(unaccented) – Eng(unaccented) contrast suggests that the results found are not specific to acoustic properties inherent in accented speech. It should be noted that no significant activity was found in the STG/S, which is thought to be involved with auditory-based speech processing.

It should be acknowledged that difference in the number of men and women in the Eng and the Jpn groups may be responsible for the between-group differences reported here. However, the Eng (Accented – Unaccented) – Jpn (Accented – Unaccented) should control for such subject differences. As well, we believe that it is unlikely that gender differences between the groups contributed to our results, given that Callan et al. (2004a) did not find gender differences using a very similar task. In addition, no gender differences were found in another study that employed speech production tasks (Buckner et al., 1995).

It has been previously suggested that activity in speech motor regions (PMC and Broca's area) may not be involved with speech intelligibility, but rather reflect differences in cognitive processes related to task difficulty, such as attention and working memory (Hickok and Poeppel, 2007; Poeppel et al., 2008; Lotto et al., 2009; Scott et al., 2009). While all four of the primary contrasts investigated in this study controlled for general processes related to the phonetic categorization task, only the contrast Jpn(accented) – Eng(accented) adequately controlled for task difficulty. The other two primary contrasts of interest [Jpn(unaccented) – Eng(unaccented) and Eng(accented-unaccented) – Jpn(accented-unaccented)] did not.

Pertinent to the issue of controlling for extraneous brain activity related to aspects of task difficulty, the four primary contrasts in this study were analyzed using phonetic identification performance as a covariate of non-interest. The results (see Tables 3, 4) showed that many of the same regions (including the PMC, Broca's area, and the cerebellum) were still found to be differentially active when performance was used as a covariate of non-interest. One drawback of using phonetic identification performance as a covariate of non-interest to control for task difficulty is that brain activity related to the processes of enhancing speech perception is likely removed by the analysis.

Of particular interest is the finding that while the perception of foreign-accented productions of a first language is related to increased activity in left PMvi/Broca's area and the right cerebellum, brain regions involved in the perception of foreign-accented productions of a second language differentially activate right PMvs/PMd and the left cerebellum instead. While left PMvi/Broca's area is thought to be involved with articulatory and sensory aspects of phonetic processing (Guenther and Vladusich, 2012), the right premotor cortex is thought to be involved with articulatory-to-auditory mapping for feedback control (Tourville and Guenther, 2011). These results are consistent with the hypothesis that the establishment of non-native phonetic categories (when the second-language is acquired after childhood) involves greater reliance on general articulatory-to-auditory feedback control systems. These systems are thought to be instantiated in right hemisphere PMC, and generate auditory predictions based on articulatory planning (Tourville and Guenther, 2011; Guenther and Vladusich, 2012).

Selective activity in right PMC and the left cerebellum (cerebellar cortical anatomical connectivity is predominantly crossed) is consistent with the hypothesis that internal models in the non-dominant hemisphere are utilized more extensively under conditions in which there is interference between established categorical representations and new representations during processing. Some additional evidence consistent with this hypothesis comes from studies in which non-native speech training led to enhanced activity in right PMC and Broca's area (Callan et al., 2003b; Wang et al., 2003; Golestani and Zatorre, 2004) and the left cerebellum (Callan et al., 2003b). Also consistent are the results of some studies investigating second-language processing that showed greater differential activity for second-language processing than for first-language processing in right PMC and Broca's area (Dehaene et al., 1997; Pillai et al., 2003) and the left cerebellum (Pillai et al., 2004). However, there are several studies that do not show any difference in brain activity between first- and second-language processing (Klein et al., 1995; Chee et al., 1999; Illes et al., 1999). It is important to note that even though the results of this study support the hypothesis that right Broca's area and the left cerebellum are differentially involved in the processing of foreign-accented productions of a second language, left Broca's area and the right cerebellum are involved with general processing of foreign-accented phonemes for both first- and second-language listeners (see Tables 3, 4). Although it is thought that the activity in the left cerebellum and right Broca's area represents articulatory-auditory internal models, it is possible that the activity represents articulatory-orosensory internal models or both articulatory-auditory and articulatory-orosensory internal models. Further experiments are needed to discern the types of internal models used under differing conditions.

The activation in left and right cerebellar lobule VI was within the region known to be involved with lip and tongue representation (Grodd et al., 2001). Given the predominantly crossed anatomical connectivity between the cerebellum and cortical areas, the finding of left PMC and right cerebellar activity that was found is consistent with the use of internal models for processing first-language phonemes. In contrast, the right PMC and left cerebellar activity that was found is consistent with the use of internal models used differentially for perception of foreign-accented productions of a second language. These results are consistent with crossed patterns of functional connectivity from the cerebellum to Broca's area that have been associated with tool use (Tamada et al., 1999). This region of the cerebellum has also been identified to be involved with speech perception and production in other studies (Ackermann et al., 2004; Callan et al., 2004a).

The finding of cerebellar activity involved in the perception of foreign-accented speech is consistent with a recent study that showed greater activity in the cerebellum after adaptation to acoustically distorted speech (Guediche et al., 2014). In contrast to our hypotheses concerning the use of forward and inverse (articulatory-auditory) internal models, Guediche et al. (2014) concluded that the cerebellum utilizes supervised learning mechanisms that rely purely on sensory prediction error signals for speech perception.

Another potential explanation of the results differentiating between processing of foreign-accented speech between first- and second-language speakers could be that there is recruitment of extra neural resources when undertaking tasks for which we are not trained. It has been shown, for example, that experienced singers, in which much of the processing is automated, show reduced activity relative to non-experienced singers (Wilson et al., 2011). It is unlikely that the results of our study can be explained by differences in task training and expertise, as the foreign-accented speech was difficult for both the English and Japanese groups, and the subjects had the same amount of training on the phonetic categorization task. As well, there was no significant difference in behavioral performance between the two groups (see Figure 1). However, it may be the case that very different processes are recruited when distinct phonetic categories exists (first-language perception), vs. when they do not (second-language perception). Although our results are consistent with the hypothesis that the establishment of second-language phonetic categories involves general articulatory-to-auditory feedback control systems in right hemisphere PMC—which generate auditory predictions based on articulatory planning, it cannot be ruled out that the pattern of differential activity reflects meta-cognitive processing strategies that result from the task requirement to identify phonetic categories that either are either from one's first or second-language. The processes may be more automatic for native speakers (or speakers with well-established phonetic categories) than for non-native speakers.

## Conclusion

The results of this study suggest that perception of foreign-accented phonetic categories involves brain regions that support aspects of speech motor control. For perception of foreign-accented productions of a first language, the activation in left PMvi/Broca's area, right cerebellum lobule VI, and the pre-SMA are consistent with the hypothesis that internal models instantiating auditory-articulatory mappings of phonemes are selected to facilitate perception. Brain regions selective for perception of second-language phonetic categories include right PMvi/Broca's, right PMvs/PMd, and the left cerebellum and are consistent with the hypothesis that articulatory-to-auditory mappings used for feedback control of speech production are used to facilitate phonetic identification. The lack of activity in the STG/S for any of the contrasts under investigation would tend to refute the hypotheses that strong engagement of bottom-up auditory processing facilitates speech perception of foreign-accented speech under these conditions. Brain regions involved with articulatory-auditory feedback for speech motor control may be a precursor for development of perceptual categories.

### Conflict of interest statement

The authors declare that the research was conducted in the absence of any commercial or financial relationships that could be construed as a potential conflict of interest.

## References

[B1] Ackermann H. Mathiak K. Ivry R. (2004). Temporal organization of “internal Speech” as a basis for cerebellar modulation of cognitive functions. Behav. Cogn. Neurosci. Rev. 3, 14–22 10.1177/1534582304263251 15191639

[B2] Adank P. Rueschemeyer S. Bekkering H. (2013). The role of accent imitation in sensorimotor integration during processing of intelligible speech. Front. Hum. Neurosci. 7:634 10.3389/fnhum.2013.00634 24109447 PMC3789941

[B3] Akahane-Yamada R. (1996). Learning non-native speech contrasts: what laboratory training studies tell us, in Proceedings of Acoustical Society of America and Acoustical Society of Japan Third Joint Meeting (Honolulu, HI), 953–958

[B4] Alho J. Sato M. Sams M. Schwartz J. Tiitinen H. Jaaskelainen I. (2012). Enhanced early-latency electromagnetic activity in the left premotor cortex is associated with successful phonetic categorization. Neuroimage 60, 1937–1946 10.1016/j.neuroimage.2012.02.011 22361165

[B5] Arbib M. (2010). Mirror system activity for action and language is embedded in the integraton of dorsal and ventral pathways. Brain Lang. 112, 12–24 10.1016/j.bandl.2009.10.001 19942271

[B6] Bradlow A. Akahane-Yamada R. Pisoni D. B. Tohkura Y. (1999). Training Japanese listeners to identify English /r/ and /l/: long-term retention of learning in perception and production. Percept. Psychophys. 61, 977–985 10.3758/BF03206911 10499009 PMC3472521

[B7] Bradlow A. Pisoni D. Akahane-Yamada R. Tohkura Y. (1997). Training Japanese listeners to identify English /r/ an /l/: IV. some effects of perceptual learning on speech production. J. Acoust. Soc. Am. 101, 2299–2310 10.1121/1.418276 9104031 PMC3507383

[B8] Buckner R. L. Raichle M. E. Petersen S. E. (1995). Dissociation of human prefrontal cortical areas across different speech production tasks and gender groups. J. Neurophysiol. 74, 2163–2173 8592204 10.1152/jn.1995.74.5.2163

[B9] Callan A. Callan D. Tajima K. Akahane-Yamada R. (2006a). Neural processes involved with perception of non-native durational contrasts. Neuroreport 17, 1353–1357 10.1097/01.wnr.0000224774.66904.29 16951584

[B10] Callan D. Callan A. Gamez M. Sato M. Kawato M. (2010). Premotor cortex mediates perceptual performance. Neuroimage 51, 844–858 10.1016/j.neuroimage.2010.02.027 20184959

[B11] Callan D. E. Callan A. M. Honda K. Masaki S. (2000). Single-sweep EEG analysis of neural processes underlying perception and production of vowels. Cogn. Brain Res. 10, 173–176 10.1016/S0926-6410(00)00025-2 10978705

[B12] Callan D. E. Jones J. A. Callan A. M. Akahane-Yamada R. (2004a). Phonetic perceptual identification by native- and second-language speakers differentially activates brain regions involved with acoustic phonetic processing and those involved with articulatory-auditory/orosensory internal models. Neuroimage 22, 1182–1194 10.1016/j.neuroimage.2004.03.006 15219590

[B13] Callan D. E. Tajima K. Callan A. M. Kubo R. Masaki S. Akahane-Yamada R. (2003b). Learning-induced neural plasticity associated with improved identification performance after training of a difficult second-language phonetic contrast. Neuroimage 19, 113–124 10.1016/S1053-8119(03)00020-X 12781731

[B14] Callan D. Jones J. A. Callan A. (2014). Multisensory and modality specific processing of visual speech in different regions of the premotor cortex. Front. Psychol. 5:389 10.3389/fpsyg.2014.00389 24860526 PMC4017150

[B15] Callan D. Jones J. A. Munhall K. Callan A. Kroos C. Vatikiotis-Bateson E. (2003a). Neural processes underlying perceptual enhancement by visual speech gestures. Neuroreport 14, 2213–2218 10.1097/01.wnr.0000095492.38740.8f 14625450

[B16] Callan D. Jones J. A. Munhall K. Kroos C. Callan A. Vatikiotis-Bateson E. (2004b). Multisensory integration sites identified by perception of spatial wavelet filtered visual speech gesture information. J. Cogn. Neurosci. 16, 805–816 10.1162/089892904970771 15200708

[B17] Callan D. Kawato M. Parsons L. Turner R. (2007). Speech and song: the role of the cerebellum. Cerebellum 6, 321–327 10.1080/14734220601187733 17853077

[B18] Callan D. Manto M. (2013). Cerebellar control of speech and song, in Handbook of the Cerebellum and Cerebellar Disorders, eds Manto M. Gruol D. Schmahmann J. Koibuchi N. Rossi F. (New York, NY: Springer), 1191–1199

[B19] Callan D. Tsytsarev V. Hanakawa T. Callan A. Katsuhara M. Fukuyama H. (2006b). Song and speech: brain regions involved with perception and covert production. Neuroimage 31, 1327–1342 10.1016/j.neuroimage.2006.01.036 16546406

[B20] Chee M. W. Tan E. W. Thiel R. (1999). Mandarin and English single word processing studied with functional magnetic resonance imaging. J. Neurosci. 19, 3050–3056 10191322 10.1523/JNEUROSCI.19-08-03050.1999 PMC6782281

[B21] Davachi L. Maril A. Wagner A. D. (2001). When keeping in mind supports later bringing to mind: neural markers of phonologi- cal rehearsal predict subsequent remembering. J. Cogn. Neurosci. 13, 1059–1070 10.1162/089892901753294356 11784444

[B22] Dehaene S. Dupoux E. Mehler J. Cohen L. Paulesu E. Perani D. (1997). Anatomical variability in the cortical representation of first and second language. Neuroreport 8, 3809–3815 10.1097/00001756-199712010-00030 9427375

[B23] Deiber M. Honda M. Ibanez V. Sadato N. Hallett M. (1999). Mesial motor areas in self-initiated versus externally triggered movements examined with fMRI: effect of movement type and rate. J. Neurophysiol. 81, 3065–3077 10368421 10.1152/jn.1999.81.6.3065

[B24] Demonet J. F. Chollet F. Ramsay S. Cardebat D. Nespoulous J. L. Wise R. S. (1992). The anatomy of phonological and semantic processing in normal subjects. Brain 115, 1753–1768 10.1093/brain/115.6.1753 1486459

[B25] Golestani N. Zatorre R. J. (2004). Learning new sounds of speech: reallocation of neural substrates. Neuroimage 21, 494–506 10.1016/j.neuroimage.2003.09.071 14980552

[B25a] Goslin J. Duffy H. Floccia C. (2012). An ERP investigation of regional and foreign accent processing. Brain Lang 122, 92–102 10.1016/j.bandl.2012.04.017 22694999

[B26] Grodd W. Hulsmann E. Lotze M. Wildgruber D. Erb M. (2001). Sensorimotor mapping of the human cerebellum: fMRI evidence of somatotopic organization. Hum. Brain Mapp. 13, 55–73 10.1002/hbm.1025 11346886 PMC6871814

[B27] Guediche S. Holt L. Laurent P. Lim S. Fiez J. (2014). Evidence for cerebellar contributions to adaptive plasticity in speech perception. Cereb. Cortex. [Epub ahead of print]. 10.1093/cercor/bht428 24451660 PMC4481605

[B28] Guenther F. Vladusich T. (2012). A neural theory of speech acquisition and production. J. Neurolinguistics 25, 408–422 10.1016/j.jneuroling.2009.08.006 22711978 PMC3375605

[B29] Hickok G. Poeppel D. (2007). The cortical organization of speech processing. Nat. Rev. Neurosci. 8, 393–402 10.1038/nrn2113 17431404

[B30] Iacoboni M. (2008). The role of premotor cortex in speech perception: evidence from fMRI and rTMS. J. Physiol. Paris 102, 31–34 10.1016/j.jphysparis.2008.03.003 18440208

[B31] Iacoboni M. Wilson S. (2006). Beyond a single area: motor control and language within a neural architecture encompassing Broca's area. Cortex 42, 503–506 10.1016/S0010-9452(08)70387-3 16881259

[B32] Illes J. Francis W. S. Desmond J. E. Gabrieli J. D. Glover G. H. Poldrack R. (1999). Convergent cortical representation of semantic processing in bilinguals. Brain Lang. 70, 347–363 10.1006/brln.1999.2186 10600225

[B33] Imamizu H. Miyauchi S. Tamada T. Sasaki Y. Takino R. Putz B. (2000). Human cerebellar activity reflecting an acquired internal model of a new tool. Nature 403, 192–195 10.1038/35003194 10646603

[B34] Jonides J. Schumacher E. H. Smith E. E. Koeppe R. A. Awh E. Reu- ter-Lorenz P. A. (1998). The role of pa- rietal cortex in verbal working memory. J. Neurosci. 18, 5026–5034 9634568 10.1523/JNEUROSCI.18-13-05026.1998 PMC6792554

[B35] Kawato M. (1999). Internal models for motor control and trajectory planning. Curr. Opin. Neurobiol. 9, 718–727 10.1016/S0959-4388(99)00028-8 10607637

[B36] Klein D. Milner B. Zatorre R. J. Meyer E. Evans A. C. (1995). The neural substrates underlying word generation: a bilingual functional imaging study. Proc. Nat. Acad. Sci. U.S.A. 92, 2899–2903 10.1073/pnas.92.7.2899 7708745 PMC42326

[B37] Lau H. C. Rogers R. D. Ramnani N. Passingham R. E. (2004). Willed action and attention to the selection of action. Neuroimage 21, 1407–1415 10.1016/j.neuroimage.2003.10.034 15050566

[B38] Lively S. Pisoni D. Yamada R. Tohkura Y. Yamada T. (1994). Training Japanese listeners to identify English /r/ and /l/. III. Long-term retention of new phonetic categories. J. Acoust. Soc. Am. 96, 2076–2087 10.1121/1.410149 7963022 PMC3518835

[B39] Lotto A. Hickok G. Holt L. (2009). Reflections on mirror neurons and speech perception. Trends Cogn. Sci. 13, 110–114 10.1016/j.tics.2008.11.008 19223222 PMC2921844

[B40] Meister I. Wilson S. Deblieck C. Wu A. Iacoboni M. (2007). The essential role of premotor cortex in speech perception. Curr. Biol. 17, 1692–1696 10.1016/j.cub.2007.08.064 17900904 PMC5536895

[B41] Miyawaki K. Strange W. Verbrugge R. Liberman A. Jenkins J. J. Fujimura O. (1975). An effect of linguistic experience: the discrimination of [r] and [l] by native speakers of Japanese and English. Percept. Psychophys. 18, 331–340 10.3758/BF03211209

[B42] Moulin-Frier C. Arbib M. (2013). Recognizing speech in a novel accent: the motor teory of speech perception reframed. Biol Cybern. 107, 421–447 10.1007/s00422-013-0557-3 23754133

[B43] Nishitani N. Schurmann M. Amunts K. Hari R. (2005). Broca's region: from action to language. Physiology 20, 60–69 10.1152/physiol.00043.2004 15653841

[B45] Pillai J. J. Allison J. D. Sethuraman S. Araque J. M. Thiruvaiyaru D. Ison C. B. (2004). Functional MR imaging study of language-related differences in bilingual cerebellar activation. Am. J. Neuroradiol. 25, 523–532 10.1016/S1053-8119(03)00151-4 15090336 PMC7975607

[B46] Pillai J. J. Araque J. M. Allison J. D. Sethuraman S. Loring D. W. Thiruvaiyaru D. (2003). Functional MRI study of semantic and phonological language processing in bilingual subjects: preliminary findings. Neuroimage 19, 565–576 10.1016/S1053-8119(03)00151-4 12880788

[B47] Poeppel D. Idsardi W. J. van Wassenhove V. (2008). Speech perception at the interface of neurobiology and linguistics. Philos. Trans. R. Soc. Lond. B Biol. Sci. 12, 363, 1071–86 10.1098/rstb.2007.2160 17890189 PMC2606797

[B48] Poldrack R. (2000). Imaging brain plasticity: conceptual and methodological issues – a theoretical review. Neuroimage 12, 1–13 10.1006/nimg.2000.0596 10875897

[B49] Price C. J. Wise R. J. Warburton E. A. Moore C. J. Howard D. Patterson K. (1996). Hearing and saying: the functional neuro-anatomy of auditory word processing. Brain 119, 919–931 10.1093/brain/119.3.919 8673502

[B50] Rauschecker J. (2011). An expanded role fore the dorsal auditory pathway in sensorimotor control and integration. Hear. Res. 271, 16–25 10.1016/j.heares.2010.09.001 20850511 PMC3021714

[B51] Rauschecker J. Scott S. (2009). Maps and streams in the auditory cortex: nonhuman primates illuminate human speech processing. Nat. Neurosci. 12, 718–724 10.1038/nn.2331 19471271 PMC2846110

[B52] Rushworth M. F. S. Walton M. E. Kennerley S. W. Bannerman D. M. (2004). Action sets and decisions in the medial frontal cortex. Trends Cogn. Sci. 8, 410–417 10.1016/j.tics.2004.07.009 15350242

[B53] Sato M. Tremblay P. Gracco V. (2009). A mediating role of the premotor cortex in phoneme segmentation. Brain Lang. 111, 1–7 10.1016/j.bandl.2009.03.002 19362734

[B54] Schmahmann J. Doyon J. Toga A. W. Petrides M. Evans A. C. (2000). MRI Atlas of the Human Cerebellum. San Diego, CA: Academic Press

[B55] Schwartz J. Basirat A. Menard L. Sato M. (2012). The perception-for-action-control theory (PACT): a perceptuo-motor theory of speech perception. J. Neurolinguist. 25, 336–354 10.1016/j.jneuroling.2009.12.004

[B55a] Scott S. K. McGettigan C. Eisner F. (2009). A little more conversation, a little less action-candidate roles for the motor cortex in speech perception. Nat. Rev. Neurosci. 10, 295–302 10.1038/nrn2603 19277052 PMC4238059

[B56] Skipper J. Goldin-Meadow S. Nusbaum H. Small S. (2007). Speech-associated gestures, Broca's area, and the human mirror system. Brain Lang. 101, 260–277 10.1016/j.bandl.2007.02.008 17533001 PMC2703472

[B57] Stevens K. (2002). Toward a model for lexical access based on acoustic landmarks and distinctive features. J. Acoust. Soc. Am. 111, 1872–1891 10.1121/1.1458026 12002871

[B58] Strange W. Jenkins J. J. (1978). Role of linguistic experience in the perception of speech, in Perception and Experience, eds Walk R. D. Pick H. L. (New York, NY: Academic), 125–169

[B59] Talairach J. Tournoux P. (1988). Co-planar Stereotactic Atlas of the Human Brain. New York, NY: Thieme

[B60] Tamada T. Miyauchi S. Imamizu H. Yoshioka T. Kawato M. (1999). Cerebro-cerebellar functional connectivity revealed by the laterality index in tool-use learning. Neuroreport 10, 325–331 10.1097/00001756-199902050-00022 10203330

[B61] Tourville J. Guenther F. (2011). The DIVA model: a neural theory of speech acquisition and production. Lang. Cogn. Process. 26, 952–981 10.1080/01690960903498424 23667281 PMC3650855

[B62] Trehub S. E. (1976). The discrimination of foreign speech contrasts by infants and adults. Child Dev. 47, 466–472 10.2307/1128803

[B63] Wang Y. Sereno J. A. Jongman A. Hirsch J. (2003). fMRI Evidence for cortical modification during learning of mandarin lexical tone. J. Cogn. Neurosci. 15, 1019–1027 10.1162/089892903770007407 14614812

[B64] Werker J. F. Gilbert J. H. V. Humphrey K. Tees R. C. (1981). Developmental aspects of cross-language speech perception. Child Dev. 52, 349–355 10.2307/1129249 7238150

[B65] Werker J. F. Tees R. C. (1999). Influences on infant speech processing: toward a new synthesis. Annu. Rev. Psychol. 50, 509–535 10.1146/annurev.psych.50.1.509 10074686

[B66] Wilson S. J. Abbott D. Lusher D. Gentle E. Jackson G. (2011). Finding your voice: a singing lesson from functional imaging. Hum. Brain Mapp. 32, 2115–2130 10.1002/hbm.21173 21162043 PMC6870391

[B67] Wilson S. M. Iacoboni M. (2006). Neural responses to non-native phonemes varying in producibility: evidence for the sensorimotor nature of speech perception. Neuroimage 33, 316–325 10.1016/j.neuroimage.2006.05.032 16919478

[B68] Wilson S. M. Saygin A. P. Sereno M. I. Iacoboni M. (2004). Listening to speech activates motor areas involved in speech production. Nat. Neurosci. 7, 701–702 10.1038/nn1263 15184903

